# Death of an Emperor

**Published:** 1878-01

**Authors:** 


### DEATH OF AN EMPEROR.

Nicholas of Russia, the Autocrat of sixty millions of subjects, was a Christian, as would seem from incidents occurring in his last and brief illness, the authority being from a source very near the throne.

"Towards night the last glimmer of a chance of recovery disappeared. On being made acquainted with the fact, the Empress, like a Christian wife, went to his bedside promptly, to impart the important intelligence to her dying husband, he being all unconscious that he was even dangerously ill. The Emperor was not prepared for this visit. She bent down over his bed and said to him tenderly and gently." It should be remembered here that, a few days before, while partaking of the Holy Sacrament, a feeling of debility or exhaustion came over the Emperor, so that he could not proceed with it. Under these circumstances, it is wonderful with what adroitness and affectionate delicacy and presence of mind the Empress made use of the fact. "My dear, you were not able to finish your devotions, so as to commune together with us, as on former occasions. Why should you not do it now? You know that for a Christian there is no better medicine, and many have even been recovered by it from their sickness."

"How!" replied the Emperor quickly, "would you have me commune in bed? I am always happy, always desirous to perform this duty. Am I then in danger?"

The Empress embraced him, and asked:

"Do you love me now as ever? as in old time?"

"Love you? yes! How should I not love you? The day we saw one another for the first time, my heart said to me, There is one who is to be your guardian angel throughout life, and this prediction of my heart has been accomplished. You are weeping!" Her tears flowed apace, and she began to repeat the Lord's Prayer in a low tone. The Emperor followed the words, and added, in a firm voice, when the Empress pronounced, "Thy will be done!". "Yes, in all things and always."

The Emperor understanding all, turned a firm and scrutinizing look on the physician, saying:

"Tell me, then, what is it? Am I dying?"

Choked with emotion, the physician said: "Yes, sire!"

After a brief silence, the Emperor inquired:

"What have you discovered in me with your stethescope? Abscesses?"

"No; but the commencement of paralysis of the lung."

"And you have had the courage, thereupon, to pronounce my sentence—to condemn me definitively to death?"

His physician reminded him that he acted in accordance with a promise exacted by the Emperor a year and a half before, when the Emperor said to him:

"I require of you to tell me the whole truth, and in time, when you see that there is need."

The Emperor listened with calm attention, and replied: "I thank you."

At the Emperor's request, the family were called in. After prayers, the Emperor said, "I pray the Lord to receive me in his arms," and partook of the Sacrament with the utmost calmness and devout fervor. After having first rendered to God the things that are God's, Nicholas next turned his attention to the affairs of his vast empire. He ordered telegrams to the chief cities to say, "The Emperor is dying," adding, "The Emperor bids adieu to Moscow." He ordered his funeral to be conducted with as little expense as possible, as it would fall ultimately on his people.

He spoke or sent kind words of remembrance to every member of his immediate household, not forgetting a child or a grandchild. But his tenderest and most constant attention was centered on her who had so long traversed with him the vicissitudes of life and empire. Addressing her, and pointing to his children present, he said: "You must live for them." And to his children: "Live always, as now, in the closest union of family affection."

A courier arrived from the Crimea, bringing letters and dispatches from his sons, the Grand Dukes Nicholas and Michael "Are they well?" inquired the Emperor. "The rest is nothing to me now. I belong wholly to God."

He then asked his physician with a smile:

"When do you mean to let me go?"

"Not yet."

"Shall I not wander, or become insensible?"

"I hope, sire, that all will pass quietly."

Then embracing his son and successor, the present Emperor, he said:

"I could have wished to have taken upon myself all that is difficult and painful, and to have left you an empire at peace, happy and flourishing. Providence has ordered it otherwise. Now I go to pray in the other world for Russia, and for you, who are, after Russia, that which I have most loved in this world."

When, having no longer strength to speak, he made a gesture between his almoner and his successor and the Empress, with what meaning must be left to conjecture; most likely it was: "Stand by one another." From that time he held their hands in his, pressing them from moment to moment, until he ceased to breathe.

From the last will of Nicholas the First, every affectionate family may learn a practical lesson of great value, in all cases, showing what a close affection existed in the royal household. It was written nearly eleven years before, to wit, on Ascension Day, May 4, 1844.

The great Autocrat, whose word and will were law to sixty million souls, seems, in the contemplation of death, although in perfect health, to have divested himself of all factitious greatness, and to have felt he was but a man; so he entitled the document his "Last Wishes." After the invocation to God the Father, God the Son, and God the Holy Ghost, come these words:

"In June, 1831, at the time of the breaking out of the cholera, I wrote down hastily my last wishes. The Lord in his mercy has not only been pleased to preserve my family, but further to permit that, since then, it should be considerably increased. These happy circumstances are of a nature to change, in part, my intentions. I desire that my wife be left in possession, for her use, of her apartments in the Winter Palace, of the Palace of Jelagin, and in the New Palace. Further, although by right of inheritance the Amtchkoff Palace belongs to my eldest son, I leave it to my wife, to enjoy it during her life, if she pleases.

"I adjure and beseech my children and grand-children to love and honor their mother, to be attentive to her comfort, to anticipate all her wishes, and to strive to console her

